# Antimicrobial resistant and extended‐spectrum β‐lactamase producing *Escherichia coli* in common wild bird species in Switzerland

**DOI:** 10.1002/mbo3.845

**Published:** 2019-04-21

**Authors:** Katrin Zurfluh, Sarah Albini, Prisca Mattmann, Patrick Kindle, Magdalena Nüesch‐Inderbinen, Roger Stephan, Barbara R. Vogler

**Affiliations:** ^1^ Vetsuisse Faculty National Centre for Enteropathogenic Bacteria and Listeria (NENT) Institute for Food Safety and Hygiene University of Zurich Zurich Switzerland; ^2^ Vetsuisse Faculty National Reference Centre for Poultry and Rabbit Diseases (NRGK) Institute for Food Safety and Hygiene University of Zurich Zurich Switzerland; ^3^ Swiss Ornithological Institute Sempach Switzerland

**Keywords:** antimicrobial resistance, ESBL, *Escherichia coli*, wild birds

## Abstract

A total of 294 fecal swabs from 294 wild birds in Switzerland were cultivated for antimicrobial resistant (AMR) *Escherichia coli*. Samples were also subcultivated to detect *E. coli* with extended‐spectrum β‐lactamases (ESBL), carbapenemases, and plasmid‐mediated aminoglycoside or colistin resistance, respectively. Samples from 17 (5.8%) of the birds contained 19 AMR *E. coli*, whereof 26.3% were multidrug resistant. Five (1.7%) ESBL‐producing *E. coli* were detected. The isolates harbored *bla*_CTX‐M‐1_ (two isolated from carrion crows and from one great spotted woodpecker, respectively), *bla*_CTX‐M‐15_ (originating from a grey heron), *bla*_CTX‐M‐55_ (from a carrion crow), and *bla*_CTX‐M‐65_ (from a common blackbird). Phylogenetic analysis assigned three isolates to commensal phylogroups A and B1, one to extraintestinal pathogenic group B2, and one to phylogroup F. Multilocus sequence typing identified sequence types (STs) that have been found previously in ESBL *E. coli* in wild birds (ST58, ST205, ST540). One isolate harboring *bla*_CTX‐M‐55_ was assigned to the recently emerged fluoroquinolone‐resistant, extraintestinal pathogenic *E. coli* clone ST1193. Wild birds have the potential to disperse AMR, including clinically important resistance genes, from anthropogenic‐influenced habitats to diverse areas, including vulnerable natural environments such as surface waters or mountain regions.

## Abstract 2

Of 294 fecal samples from resident and short‐distance migratory wild birds, 5.8% contained antimicrobial resistant (AMR) *Escherichia coli*. Moreover, 1.7% of the samples yielded *E. coli* producing the extended‐spectrum β‐lactamases CTX‐M‐1, CTX‐M‐15, CTX‐M‐55, or CTX‐M‐65. Wild birds have the potential to disperse AMR bacteria from anthropogenic‐influenced habitats to diverse areas, including vulnerable natural environments such as surface waters or mountain regions.

Antimicrobial resistance (AMR) is one of the major threats to public health. AMR affects humans, animals, and the environment and intervention requires a one health approach (World Health Organization, 2014). Although wildlife is rarely directly exposed to antimicrobial agents, wild birds may be colonized or infected with resistant bacteria (Guenther, Ewers, & Wieler, 2011; Oteo et al., 2018). The emergence of AMR in wild birds is thought to be due to the dissemination of resistant bacteria or AMR genes from anthropogenic‐influenced habitats to the natural environment via contaminated water or feed (Guenther et al., 2010). In turn, wild birds may represent a reservoir for bacteria harboring clinically important resistance determinants (Arnold, Williams, & Bennett, 2016). Migratory wild birds in particular, are known to be disseminators of AMR into geographically distant ecosystems (Agnew, Wang, Fanning, Bearhop, & McMahon, 2015). There is a growing number of reports of bacteria with clinically relevant AMR isolated from wild birds worldwide, including strains harboring extended‐spectrum β‐lactamase (ESBL) genes (*bla*_ESBL_), carbapenemase genes, aminoglycoside, and quinolone resistance genes (Bonnedahl et al., 2010; Guenther et al., 2012; Oteo et al., 2018). By contrast, information on the occurrence of AMR in wild and peri‐domesticated birds in Switzerland is scarce. In one study, feral pigeons and great cormorants were identified as potential reservoirs of ESBL‐producing *Escherichia* (*E*.) *coli* (Zurfluh, Nüesch‐Inderbinen, Stephan, & Hächler, 2013). However, there is a lack of data regarding the occurrence of AMR among other wild birds, including resident and migratory birds.

The Swiss Ornithological Institute in Sempach is a nongovernmental organization that monitors the populations of breeding birds, migrants, and winter visitors in Switzerland. In addition, it maintains a rescue, care, and rehabilitation center for orphaned or injured wild birds. In this study, we aimed to investigate fecal samples from wild birds admitted to the care center for the occurrence of AMR *E. coli*, and to identify *Enterobacteriaceae* with transmissible resistance to third‐generation cephalosporins, carbapenems, aminoglycosides, and colistin.

Between May and October 2018, fecal swabs were collected in the rehabilitation center from 294 birds representing 55 different species. The majority (246/83.7%) were nestlings, pulli, or juvenile birds. A complete list of the birds is available in Appendix Table A1 From live birds, swabs of freshly passed feces were sampled directly from a sterile surface, and from dead birds a fecal swab was collected from the cloaca. Swabs were numbered consecutively and admitted to the laboratory for analysis. For nonselective isolation of *E. coli*, swabs were inoculated onto Rapid’*E. coli* 2 Agar Plates (Bio‐Rad Laboratories, Reinach, Switzerland). For selective isolation of resistant *Enterobacteriaceae*, the same swabs were thereafter placed in 5 ml tubes containing *Enterobacteriaceae* Enrichment broth (BD, Franklin Lakes) and incubated at 37°C for 24 hr. One loopful of each enrichment was inoculated onto Brilliance ESBL Agar (Oxoid/Thermo Fisher Scientific, Waltham, MA), chromID^®^ CARBA SMART (bioMérieux, Marcy l'Etoile, France), amikacin‐Luria Bertani agar (200 mg/L amikacin, 10 mg/ml vancomycin, and 5 mg/L amphotericin B), and colistin‐bromothymol blue lactose agar (4 mg/L colistin, 10 mg/ml vancomycin, and 5 mg/L amphotericin B). Plates were incubated at 37°C for 24 hr. Species identity was confirmed by matrix‐assisted laser desorption/ionization time‐of‐flight mass spectrometry (Biotyper^®^ MALDI‐TOF‐MS system by Bruker Daltonics, Billerica, MA) using Compass FlexControl version 3.4 software with the Compass database version 4.1.80.

From 158 fecal samples (53.7% of all samples), a total of 256 *E. coli* isolates were obtained by nonselective cultivation. Antimicrobial susceptibility testing using the disk‐diffusion method according the Clinical and Laboratory Standards Institute performance standards (Clinical and Laboratory Standards Institute, 2017) detected antibiotic resistance in 19 (7.4%) of the *E. coli* isolates which originated from 17 (5.8%) of the 294 analyzed birds (Table 1). One sample from a feral pigeon yielded three *E. coli* strains with distinct resistance profiles (Table 1). Multidrug resistance (MDR), defined as resistance to three or more classes of antimicrobials was identified in 5 (26.3%) of the 19 resistant *E. coli* isolates, thus in 2% of the 256 isolated *E. coli* (Table 1).

**Table 1:** Antimicrobial resistant *Escherichia coli* obtained by nonselective isolation from fecal swabs of wild birds

Isolate	Resistance profile	Wild birds			
		Species	Common name	Migratory behavior	Age
191 C1	AM, CZ, TE	*Columba livia domestica*	Feral pigeon	Resident	Juvenile
273 C1	AM, TE	*Columba livia domestica*	Feral pigeon	Resident	Adult
151 C1	AM, NA, SXT, S, C, TE	*Columba livia domestica*	Feral pigeon	Resident	Juvenile
151 C2	AM, NA, CIP, SXT, AZM, F/M, S, GM, C, TE				
151 C3	AM, NA, CIP, S, K, C, TE				
070 C1	CZ, SXT	*Cygnus olor*	Mute swan	Resident	Nestling
255 C1	AM, NA, CIP, SXT, C, TE	*Streptopelia decaocto*	Eurasian collared dove	Resident	Adult
039 C1	S	*Anas platyrhynchos*	Mallard	Resident to short distance	Pullus
046 C4	AM, CZ, AMC, AZM	*Anas platyrhynchos*	Mallard	Resident to short distance	Pullus
193 C1	K	*Anas platyrhynchos*	Mallard	Resident to short distance	Juvenile
277 C1	AM, S, GM	*Buteo buteo*	Common buzzard	Resident to short distance	Adult
009 C3	CZ	*Corvus corone*	Carrion crow	Resident to short distance	Nestling
114 C1	TE	*Corvus corone*	Carrion crow	Resident to short distance	Nestling
202 C1	TE	*Larus michahellis*	Yellow‐legged gull	Resident to short distance	Juvenile
200 C1	NA, TE	*Milvus milvus*	Red kite	Resident to short distance	Juvenile
203 C1	NA	*Milvus milvus*	Red kite	Resident to short distance	Juvenile
147 C1	NA, CIP, SXT, C	*Turdus merula*	Common blackbird	Resident to short distance	Juvenile
198 C1	NA	*Falco subbuteo*	Eurasian hobby	Long distance	Adult
263 C1	TE	*Hirundo rustica*	Barn swallow	Long distance	Juvenile

Note

AM: ampicillin; AMC: amoxicillin/clavulanic acid; AZM: azithromycin; C: chloramphenicol; CIP: ciprofloxacin; CZ: cefazolin; F/M: nitrofurantoin; GM: gentamicin; K: kanamycin; NA: nalidixic acid; S: streptomycin; SXT: sulfamethoxazole/trimethoprim; TE: tetracycline.

Among the 17 birds with AMR *E. coli*, 5 (29.4%) were resident birds, 10 (58.8%) were birds with resident to short‐distance migratory behavior, and 2 (11.8%) were long‐distance migrants (Table 1). The majority (13/76.5%) were nestlings, pulli, or juvenile birds (Table 1). Their AMR *E. coli* are likely acquired via parental transfer. Therefore, it cannot be excluded that the occurrence of AMR in adult birds, which are exposed to a diverse pool of AMR, may be higher than our data suggest.

Within the set of AMR *E. coli*, high rates of resistance to ampicillin (8/19 isolates), nalidixic acid (8/19), and tetracycline (10/19) were observed. Resistance to chloramphenicol (5/19), ciprofloxacin (4/19), cefazolin (4/19), streptomycin (5/19), and sulfamethoxazole/trimethoprim (5/19) were less often detected, and resistance to amoxicillin/clavulanic acid (1/19), azithromycin (2/19), nitrofurantoin (1/19), gentamicin (2/19), and kanamycin (2/19) occurred rarely (Table 1). The high rate of resistance to ampicillin and tetracycline correlates with AMR data reported by the Federal Office of Public Health for *E. coli* isolated from livestock in Switzerland (Federal Office of Public Health and Federal Food Safety and Veterinary Office, 2018), indicating potential transmission events of AMR between livestock and wildlife.

By selective cultivation, five ESBL‐producing *E. coli* were detected in samples from five (1.7%) of the 294 birds, including two carrion crows, a common blackbird, a great spotted woodpecker, and a grey heron (Table 2). Phylogenetic groups, multilocus sequence types (STs), and *bla*_ESBL_ genes were determined by PCR and sequencing as described previously (Clermont, Christenson, Denamur, & Gordon, 2013; Wirth et al., 2006; Woodford, Fagan, & Ellington, 2006; Zurfluh, Hächler, Nüesch‐Inderbinen, & Stephan, 2013) (Table 2). Two isolates belonging to commensal phylogroups A and B1 harbored *bla*_CTX‐M‐1_. A further strain assigned to phylogroup B1 carried *bla*_CTX‐M‐65_. One isolate belonged to extraintestinal pathogenic phylogenetic group B2 and carried *bla*_CTX‐M‐55_. One isolate harboring *bla*_CTX‐M‐15_ was assigned to phylogenetic group F (Table 2). This particular phylogroup is rarely identified, but of particular interest, since strains belonging to this group are associated with extraintestinal infections of companion animals and have been frequently detected in wild birds being treated in wildlife rehabilitation centers (Vangchhia et al., 2016). Overall, five different STs were detected among the ESBL *E. coli* (Table 2). The majority of these, including ST58, ST205, ST540, and ST1722, have already been reported in ESBL‐producing *E. coli* from waterfowl in Pakistan (Mohsin et al., 2017), gulls in Chile (Hernandez et al., 2013) Portugal (Guenther et al., 2011) and Sweden (Atterby et al., 2017). Interestingly, *E. coli* ST1193 belongs to extraintestinal pathogenic pandemic clonal group associated with urinary tract infections (UTIs) in humans and is, similar to the *E. coli* ST131 H30 clone, an important contributor to fluoroquinolone resistance worldwide (Tchesnokova et al., 2019). This clone has very recently emerged as an MDR *bla*_CTX‐M‐14_ and *bla*_CTX‐M‐15_ harboring nosocomial pathogen in Germany (Valenza et al., 2019). Further, *E. coli* ST1193 harboring *bla*_CTX‐M‐55_ represents one of the most prevalent ESBL‐producing *E. coli* associated with UTI in China (Xia et al., 2017). Our results indicate that wild birds may contribute to the global dissemination of this important clonal lineage.

**Table 2:** Characteristics of extended‐spectrum β‐lactamase (ESBL)‐producing *Escherichia coli* isolates from wild birds in Switzerland

Isolate	PG	ST	ESBL	Resistance profile	Wild birds			
					Species	Common name	Migratory behavior	Age
127	A	540	CTX‐M‐1	AM, CZ, CTX, K, TE	*Dendrocopos major*	Great spotted woodpecker	Resident	Juvenile
114	B1	205	CTX‐M‐1	AM, CZ, CTX, NA, CIP, SXT, S, K, TE	*Corvus corone*	Carrion crow	Resident to short distance	Nestling
9	B2	1193	CTX‐M‐55	AM, CZ, CTX, NA, CIP, SXT, AZM, GM	*Corvus corone*	Carrion crow	Resident to short distance	Nestling
59	B1	58	CTX‐M‐65	AM, CZ, CTX, C, TE	*Turdus merula*	Common blackbird	Resident to short distance	Nestling
235	F	1722	CTX‐M‐15	AM, CZ, CTX, FEP, SXT, S, TE	*Ardea cinerea*	Grey heron	Short distance	Adult

Note

AM: ampicillin; AZM: azithromycin; C: chloramphenicol; CIP: ciprofloxacin; CTX: cefotaxime; CZ: cefazolin; FEP: cefepime; GM: gentamicin; K: kanamycin; NA: nalidixic acid; PG: phylogenetic group; S: streptomycin; ST: sequence type; SXT: sulfamethoxazole/trimethoprim; TE: tetracycline.

Using selective cultivation with colistin, two *Enterobacter asburiae* and one *Enterobacter cancerogenus* were found (data not shown). Screening for plasmid‐mediated colistin resistance genes *mcr‐1* through *mcr‐5* was undertaken by PCR (Rebelo et al., 2018), but remained negative for all isolates. Selective isolation using chromID^®^ CARBA SMART or amikacin did not yield any strains.

Of the ESBLs detected in this study, CTX‐M‐15, isolated from *E. coli* from a grey heron in this study, is one of the most important ESBLs in human medicine. *E. coli* producing this enzyme has emerged worldwide as an important cause of bloodstream infections and of community‐acquired UTIs (Cantón, González‐Alba, & Galán, 2012). Its single amino acid variant, CTX‐M‐55, identified in a sample from a carrion crow, was first detected in Thailand from *E. coli* and *Klebsiella pneumoniae* causing community‐ and hospital‐acquired infection and has since been found widely in food‐producing animals and humans in China (Zhang et al., 2014). Similarly, CTX‐M‐65 is a prevalent ESBL in animal and human *E. coli* strains and *Salmonella* spp. in China (Bai et al., 2016). By contrast, CTX‐M‐1 is prevalent within the poultry industry and other livestock in Europe (Zurfluh et al., 2014). In Switzerland, all four ESBL variants described in this study have been detected in *E. coli* isolated from healthy humans, livestock, food, surface water, and fish from Swiss lakes (Müller, Stephan, & Nüesch‐Inderbinen, 2016). Notably, the ESBL variants were all isolated from birds frequently observed in urbanized areas, thus, the occurrence of these variants in wild birds is likely associated with proximity to human and livestock‐associated settings, as well as exposure to contaminated surface waters (Guenther et al., 2012).

Reassuringly, *bla*_ESBL_ genes were rare among the birds analyzed in this study, and no acquired carbapenemase genes, *mcr* genes, or transmissible aminoglycoside genes were detected. Our results contrast with data reported in a study that identified *bla*_ESBL_ genes, the carbapenemase gene *bla*_OXA‐48_, and *mcr* in 8.7%, 0.8%, and 0.1%, respectively, of 28 species of wild birds in Spain (Oteo et al., 2018). However, caution should be used when comparing data obtained using different methods for screening for ESBL producers. In contrast to Oteo et al. (2018), we used a semi‐selective enrichment step prior to plating on selective ESBL agar, which may have affected the recovery rate. The difference in the performance of semi‐selective (using broth selecting for Gram negative rods) and selective enrichment (i.e., broth additionally containing a third‐generation cephalosporin) remains to be elucidated and comparative data are limited (Blane et al., 2016; Jazmati, Hein, & Hamprecht, 2016; Kluytmans‐Van Den Bergh et al., 2015).

Migratory birds in particular have been identified as potent disseminators of AMR, because they may cover long distances and impact diverse ecological niches by introducing new or emerging AMR (Agnew et al., 2015). Whilst the majority of the birds from this study were resident or short‐distance migratory birds, they nonetheless have the potential to disperse AMR to diverse areas, including vulnerable environments such as surface waters or mountain regions. Moreover, they can be considered indicators for the occurrence and distribution of AMR in the environment.

## CONFLICT OF INTERESTS

None declared.

## AUTHOR CONTRIBUTIONS

RS and BRV contributed to design of the study. PM performed sampling. KZ and PK performed laboratory analysis. KZ, SA, RS, BRV, and MN‐I performed data analysis. KZ and MN‐I drafted the manuscript. All authors contributed helpful comments and approved the final version of the manuscript.

## ETHICS STATEMENT

This study conformed to the legal requirements of Switzerland and in accordance with the guidelines of the Swiss Ornithological Institute.

## Data Availability

All raw data generated during the current study are available from the corresponding author on reasonable request.

## Appendix

**Table A1:** Overview of 294 wild birds screened for fecal cloacal occurrence of antimicrobial resistant *Escherichia coli*

Strain ID	Species	Common name	No. birds/species	Migratory behavior	Age
AMR 0194	*Tachymarptis melba*	Alpine swift	3	Long distance	Juvenile
AMR 0227	*Tachymarptis melba*	Alpine swift		Long distance	Juvenile
AMR 0238	*Tachymarptis melba*	Alpine swift		Long distance	Juvenile
AMR 0285	*Tyto alba*	Barn owl	1	Resident	Juvenile
AMR 0231	*Hirundo rustica*	Barn swallow	5	Long distance	Juvenile
AMR 0263	*Hirundo rustica*	Barn swallow		Long distance	Juvenile
AMR 0279	*Hirundo rustica*	Barn swallow		Long distance	Juvenile
AMR 0290	*Hirundo rustica*	Barn swallow		Long distance	Juvenile
AMR 0294	*Hirundo rustica*	Barn swallow		Long distance	Juvenile
AMR 0144	*Milvus migrans*	Black kite	1	Long distance	Adult
AMR 0026	*Phoenicurus ochruros*	Black redstart	7	Short distance	Nestling
AMR 0069	*Phoenicurus ochruros*	Black redstart		Short distance	Adult
AMR 0091	*Phoenicurus ochruros*	Black redstart		Short distance	Juvenile
AMR 0092	*Phoenicurus ochruros*	Black redstart		Short distance	Juvenile
AMR 0137	*Phoenicurus ochruros*	Black redstart		Short distance	Adult
AMR 0145	*Phoenicurus ochruros*	Black redstart		Short distance	Adult
AMR 0274	*Phoenicurus ochruros*	Black redstart		Short distance	Adult
AMR 0006	*Corvus corone*	Carrion crow	13	Resident to short distance	Nestling
AMR 0009	*Corvus corone*	Carrion crow		Resident to short distance	Nestling
AMR 0028	*Corvus corone*	Carrion crow		Resident to short distance	Juvenile
AMR 0071	*Corvus corone*	Carrion crow		Resident to short distance	Pullus
AMR 0072	*Corvus corone*	Carrion crow		Resident to short distance	Nestling
AMR 0081	*Corvus corone*	Carrion crow		Resident to short distance	Nestling
AMR 0103	*Corvus corone*	Carrion crow		Resident to short distance	Nestling
AMR 0114	*Corvus corone*	Carrion crow		Resident to short distance	Nestling
AMR 0117	*Corvus corone*	Carrion crow		Resident to short distance	Nestling
AMR 0118	*Corvus corone*	Carrion crow		Resident to short distance	Nestling
AMR 0119	*Corvus corone*	Carrion crow		Resident to short distance	Nestling
AMR 0169	*Corvus corone*	Carrion crow		Resident to short distance	Juvenile
AMR 0192	*Corvus corone*	Carrion crow		Resident to short distance	Adult
AMR 0005	*Turdus merula*	Common blackbird	45	Resident to short distance	Juvenile
AMR 0010	*Turdus merula*	Common blackbird		Resident to short distance	Juvenile
AMR 0017	*Turdus merula*	Common blackbird		Resident to short distance	Juvenile
AMR 0024	*Turdus merula*	Common blackbird		Resident to short distance	Nestling
AMR 0031	*Turdus merula*	Common blackbird		Resident to short distance	Nestling
AMR 0033	*Turdus merula*	Common blackbird		Resident to short distance	Nestling
AMR 0059	*Turdus merula*	Common blackbird		Resident to short distance	Nestling
AMR 0060	*Turdus merula*	Common blackbird		Resident to short distance	Nestling
AMR 0061	*Turdus merula*	Common blackbird		Resident to short distance	Juvenile
AMR 0064	*Turdus merula*	Common blackbird		Resident to short distance	Juvenile
AMR 0066	*Turdus merula*	Common blackbird		Resident to short distance	Nestling
AMR 0076	*Turdus merula*	Common blackbird		Resident to short distance	Juvenile
AMR 0077	*Turdus merula*	Common blackbird		Resident to short distance	Juvenile
AMR 0084	*Turdus merula*	Common blackbird		Resident to short‐distance	Nestling
AMR 0085	*Turdus merula*	Common blackbird		Resident to short distance	Nestling
AMR 0105	*Turdus merula*	Common blackbird		Resident to short distance	Pullus
AMR 0106	*Turdus merula*	Common blackbird		Resident to short distance	Nestling
AMR 0109	*Turdus merula*	Common blackbird		Resident to short distance	Adult
AMR 0115	*Turdus merula*	Common blackbird		Resident to short distance	Juvenile
AMR 0124	*Turdus merula*	Common blackbird		Resident to short distance	Juvenile
AMR 0130	*Turdus merula*	Common blackbird		Resident to short distance	Juvenile
AMR 0133	*Turdus merula*	Common blackbird		Resident to short distance	Nestling
AMR 0135	*Turdus merula*	Common blackbird		Resident to short distance	Juvenile
AMR 0143	*Turdus merula*	Common blackbird		Resident to short distance	Adult
AMR 0147	*Turdus merula*	Common blackbird		Resident to short distance	Juvenile
AMR 0154	*Turdus merula*	Common blackbird		Resident to short distance	Nestling
AMR 0161	*Turdus merula*	Common blackbird		Resident to short distance	Nestling
AMR 0162	*Turdus merula*	Common blackbird		Resident to short distance	Nestling
AMR 0163	*Turdus merula*	Common blackbird		Resident to short distance	Nestling
AMR 0171	*Turdus merula*	Common blackbird		Resident to short distance	Nestling
AMR 0175	*Turdus merula*	Common blackbird		Resident to short distance	Nestling
AMR 0176	*Turdus merula*	Common blackbird		Resident to short distance	Nestling
AMR 0211	*Turdus merula*	Common blackbird		Resident to short distance	Nestling
AMR 0213	*Turdus merula*	Common blackbird		Resident to short‐distance	Juvenile
AMR 0223	*Turdus merula*	Common blackbird		Resident to short distance	Adult
AMR 0239	*Turdus merula*	Common blackbird		Resident to short distance	Juvenile
AMR 0240	*Turdus merula*	Common blackbird		Resident to short distance	Juvenile
AMR 0241	*Turdus merula*	Common blackbird		Resident to short distance	Juvenile
AMR 0242	*Turdus merula*	Common blackbird		Resident to short distance	Adult
AMR 0247	*Turdus merula*	Common blackbird		Resident to short distance	Juvenile
AMR 0254	*Turdus merula*	Common blackbird		Resident to short distance	Adult
AMR 0257	*Turdus merula*	Common blackbird		Resident to short distance	Juvenile
AMR 0258	*Turdus merula*	Common blackbird		Resident to short distance	Juvenile
AMR 0264	*Turdus merula*	Common blackbird		Resident to short distance	Adult
AMR 0297	*Turdus merula*	Common blackbird		Resident to short distance	Adult
AMR 0197	*Buteo buteo*	Common buzzard	2	Resident to short distance	Adult
AMR 0277	*Buteo buteo*	Common buzzard		Resident to short distance	Adult
AMR 0102	*Fringilla coelebs*	Common caffinch	4	Resident to short distance	Juvenile
AMR 0150	*Fringilla coelebs*	Common caffinch		Resident to short distance	Nestling
AMR 0220	*Fringilla coelebs*	Common caffinch		Resident to short distance	Nestling
AMR 0222	*Fringilla coelebs*	Common caffinch		Resident to short distance	Adult
AMR 0204	*Delichon urbicum*	Common house martin	3	Long distance	Adult
AMR 0205	*Delichon urbicum*	Common house martin		Long distance	Nestling
AMR 0284	*Delichon urbicum*	Common house martin		Long distance	Adult
AMR 0123	*Falco tinnunculus*	Common kestrel	6	Resident to short distance	Pullus
AMR 0146	*Falco tinnunculus*	Common kestrel		Resident to short distance	Nestling
AMR 0157	*Falco tinnunculus*	Common kestrel		Resident to short distance	Juvenile
AMR 0160	*Falco tinnunculus*	Common kestrel		Resident to short distance	Juvenile
AMR 0190	*Falco tinnunculus*	Common kestrel		Resident to short distance	Juvenile
AMR 0196	*Falco tinnunculus*	Common kestrel		Resident to short distance	Juvenile
AMR 0120	*Alcedo atthis*	Common kingfisher	1	Resident to short distance	Nestling
AMR 0011	*Mergus merganser*	Common merganser	6	Resident to short distance	Juvenile
AMR 0012	*Mergus merganser*	Common merganser		Resident to short distance	Juvenile
AMR 0013	*Mergus merganser*	Common merganser		Resident to short distance	Juvenile
AMR 0014	*Mergus merganser*	Common merganser		Resident to short distance	Juvenile
AMR 0015	*Mergus merganser*	Common merganser		Resident to short distance	Juvenile
AMR 0016	*Mergus merganser*	Common merganser		Resident to short distance	Juvenile
AMR 0030	*Sturnus vulgaris*	Common starling	3	Short distance	Nestling
AMR 0080	*Sturnus vulgaris*	Common starling		Short distance	Nestling
AMR 0281	*Sturnus vulgaris*	Common starling		Short distance	Adult
AMR 0063	*Apus apus*	Common swift	16	Long distance	Adult
AMR 0107	*Apus apus*	Common swift		Long distance	Nestling
AMR 0108	*Apus apus*	Common swift		Long distance	Adult
AMR 0149	*Apus apus*	Common swift		Long distance	Nestling
AMR 0153	*Apus apus*	Common swift		Long distance	Juvenile
AMR 0159	*Apus apus*	Common swift		Long distance	Nestling
AMR 0167	*Apus apus*	Common swift		Long distance	Juvenile
AMR 0170	*Apus apus*	Common swift		Long distance	Juvenile
AMR 0172	*Apus apus*	Common swift		Long distance	Juvenile
AMR 0173	*Apus apus*	Common swift		Long distance	Nestling
AMR 0174	*Apus apus*	Common swift		Long distance	Nestling
AMR 0182	*Apus apus*	Common swift		Long distance	Pullus
AMR 0195	*Apus apus*	Common swift		Long distance	Juvenile
AMR 0218	*Apus apus*	Common swift		Long distance	Nestling
AMR 0248	*Apus apus*	Common swift		Long distance	Juvenile
AMR 0262	*Apus apus*	Common swift		Long distance	Juvenile
AMR 0185	*Columba palumbus*	Common wood pigeon	5	Resident to short distance	Juvenile
AMR 0207	*Columba palumbus*	Common wood pigeon		Resident to short distance	Juvenile
AMR 0244	*Columba palumbus*	Common wood pigeon		Resident to short distance	Nestling
AMR 0265	*Columba palumbus*	Common wood pigeon		Resident to short distance	Adult
AMR 0283	*Columba palumbus*	Common wood pigeon		Resident to short distance	Juvenile
AMR 0089	*Sylvia atricapilla*	Eurasian blackcap	6	Short distance	Juvenile
AMR 0090	*Sylvia atricapilla*	Eurasian blackcap		Short distance	Juvenile
AMR 0093	*Sylvia atricapilla*	Eurasian blackcap		Short distance	Juvenile
AMR 0094	*Sylvia atricapilla*	Eurasian blackcap		Short distance	Juvenile
AMR 0140	*Sylvia atricapilla*	Eurasian blackcap		Short distance	Juvenile
AMR 0221	*Sylvia atricapilla*	Eurasian blackcap		Short distance	Nestling
AMR 0018	*Cyanistes caeruleus*	Eurasian blue tit	9	Resident to short distance	Nestling
AMR 0021	*Cyanistes caeruleus*	Eurasian blue tit		Resident to short distance	Nestling
AMR 0022	*Cyanistes caeruleus*	Eurasian blue tit		Resident to short distance	Nestling
AMR 0087	*Cyanistes caeruleus*	Eurasian blue tit		Resident to short distance	Nestling
AMR 0097	*Cyanistes caeruleus*	Eurasian blue tit		Resident to short distance	Nestling
AMR 0098	*Cyanistes caeruleus*	Eurasian blue tit		Resident to short distance	Juvenile
AMR 0099	*Cyanistes caeruleus*	Eurasian blue tit		Resident to short distance	Nestling
AMR 0100	*Cyanistes caeruleus*	Eurasian blue tit		Resident to short distance	Nestling
AMR 0101	*Cyanistes caeruleus*	Eurasian blue tit		Resident to short distance	Juvenile
AMR 0255	*Streptopelia decaocto*	Eurasian collared dove	4	Resident	Adult
AMR 0269	*Streptopelia decaocto*	Eurasian collared dove		Resident	Juvenile
AMR 0272	*Streptopelia decaocto*	Eurasian collared dove		Resident	Juvenile
AMR 0291	*Streptopelia decaocto*	Eurasian collared dove		Resident	Juvenile
AMR 0122	*Fulica atra*	Eurasian coot	3	Resident to short distance	Juvenile
AMR 0125	*Fulica atra*	Eurasian coot		Resident to short distance	Juvenile
AMR 0180	*Fulica atra*	Eurasian coot		Resident to short distance	Juvenile
AMR 0266	*Bubo bubo*	Eurasian eagle‐owl	1	Resident	Juvenile
AMR 0198	*Falco subbuteo*	Eurasian hobby	1	Long distance	Adult
AMR 0065	*Garrulus glandarius*	Eurasian jay	1	Resident to short distance	Adult
AMR 0003	*Pica pica*	Eurasian magpie	10	Resident	Nestling
AMR 0004	*Pica pica*	Eurasian magpie		Resident	Nestling
AMR 0068	*Pica pica*	Eurasian magpie		Resident	Nestling
AMR 0079	*Pica pica*	Eurasian magpie		Resident	Pullus
AMR 0083	*Pica pica*	Eurasian magpie		Resident	Juvenile
AMR 0129	*Pica pica*	Eurasian magpie		Resident	Juvenile
AMR 0152	*Pica pica*	Eurasian magpie		Resident	Adult
AMR 0201	*Pica pica*	Eurasian magpie		Resident	Juvenile
AMR 0206	*Pica pica*	Eurasian magpie		Resident	Nestling
AMR 0237	*Pica pica*	Eurasian magpie		Resident	Juvenile
AMR 0245	*Acrocephalus scirpaceus*	Eurasian reed warbler	1	Long distance	Juvenile
AMR 0275	*Accipiter nisus*	Eurasian sparrowhawk	1	Short distance	Juvenile
AMR 0008	*Passer montanus*	Eurasian tree sparrow	4	Resident to short distance	Nestling
AMR 0208	*Passer montanus*	Eurasian tree sparrow		Resident to short distance	Juvenile
AMR 0209	*Passer montanus*	Eurasian tree sparrow		Resident to short distance	Nestling
AMR 0210	*Passer montanus*	Eurasian tree sparrow		Resident to short distance	Nestling
AMR 0062	*Carduelis carduelis*	European goldfinch	8	Resident to short distance	Juvenile
AMR 0168	*Carduelis carduelis*	European goldfinch		Resident to short distance	Adult
AMR 0178	*Carduelis carduelis*	European goldfinch		Resident to short distance	Nestling
AMR 0217	*Carduelis carduelis*	European goldfinch		Resident to short distance	Juvenile
AMR 0219	*Carduelis carduelis*	European goldfinch		Resident to short distance	Juvenile
AMR 0243	*Carduelis carduelis*	European goldfinch		Resident to short distance	Juvenile
AMR 0250	*Carduelis carduelis*	European goldfinch		Resident to short distance	Adult
AMR 0276	*Carduelis carduelis*	European goldfinch		Resident to short distance	Juvenile
AMR 0075	*Picus viridis*	European green woodpecker	4	Resident	Juvenile
AMR 0189	*Picus viridis*	European green woodpecker		Resident	Juvenile
AMR 0252	*Picus viridis*	European green woodpecker		Resident	Adult
AMR 0287	*Picus viridis*	European green woodpecker		Resident	Juvenile
AMR 0228	*Chloris chloris*	European greenfinch	2	Resident to short distance	Juvenile
AMR 0253	*Chloris chloris*	European greenfinch		Resident to short distance	Juvenile
AMR 0261	*Ficedula hypoleuca*	European pied flycatcher	1	Long distance	Juvenile
AMR 0082	*Erithacus rubecula*	European robin	2	Short distance	Juvenile
AMR 0296	*Erithacus rubecula*	European robin		Short distance	Adult
AMR 0187	*Serinus serinus*	European serin	1	Short distance	Nestling
AMR 0112	*Columba livia domestica*	Feral pigeon	11	Resident	Nestling
AMR 0142	*Columba livia domestica*	Feral pigeon		Resident	Nestling
AMR 0151	*Columba livia domestica*	Feral pigeon		Resident	Juvenile
AMR 0165	*Columba livia domestica*	Feral pigeon		Resident	Nestling
AMR 0191	*Columba livia domestica*	Feral pigeon		Resident	Juvenile
AMR 0199	*Columba livia domestica*	Feral pigeon		Resident	Nestling
AMR 0232	*Columba livia domestica*	Feral pigeon		Resident	Juvenile
AMR 0268	*Columba livia domestica*	Feral pigeon		Resident	Juvenile
AMR 0273	*Columba livia domestica*	Feral pigeon		Resident	Adult
AMR 0286	*Columba livia domestica*	Feral pigeon		Resident	Adult
AMR 0298	*Columba livia domestica*	Feral pigeon		Resident	Nestling
AMR 0095	*Turdus pilaris*	Fieldfare	3	Short distance	Juvenile
AMR 0138	*Turdus pilaris*	Fieldfare		Short distance	Juvenile
AMR 0179	*Turdus pilaris*	Fieldfare		Short distance	Nestling
AMR 0282	*Sylvia borin*	Garden warbler	1	Long distance	Adult
AMR 0088	*Dendrocopos major*	Great spotted woodpecker	3	Resident	Juvenile
AMR 0127	*Dendrocopos major*	Great spotted woodpecker		Resident	Juvenile
AMR 0156	*Dendrocopos major*	Great spotted woodpecker		Resident	Pullus
AMR 0019	*Parus major*	Great tit	7	Resident to short distance	Nestling
AMR 0020	*Parus major*	Great tit		Resident to short distance	Nestling
AMR 0041	*Parus major*	Great tit		Resident to short distance	Nestling
AMR 0042	*Parus major*	Great tit		Resident to short distance	Nestling
AMR 0139	*Parus major*	Great tit		Resident to short distance	Nestling
AMR 0166	*Parus major*	Great tit		Resident to short distance	Adult
AMR 0225	*Parus major*	Great tit		Resident to short distance	Juvenile
AMR 0235	*Ardea cinerea*	Grey heron	2	Short distance	Adult
AMR 0260	*Ardea cinerea*	Grey heron		Short distance	Juvenile
AMR 0230	*Coccothraustes coccothraustes*	Hawfinch	1	Resident to short distance	Nestling
AMR 0002	*Passer domesticus*	House sparrow	40	Resident	Nestling
AMR 0025	*Passer domesticus*	House sparrow		Resident	Nestling
AMR 0029	*Passer domesticus*	House sparrow		Resident	Nestling
AMR 0053	*Passer domesticus*	House sparrow		Resident	Nestling
AMR 0054	*Passer domesticus*	House sparrow		Resident	Nestling
AMR 0055	*Passer domesticus*	House sparrow		Resident	Nestling
AMR 0056	*Passer domesticus*	House sparrow		Resident	Nestling
AMR 0057	*Passer domesticus*	House sparrow		Resident	Nestling
AMR 0067	*Passer domesticus*	House sparrow		Resident	Nestling
AMR 0073	*Passer domesticus*	House sparrow		Resident	Nestling
AMR 0074	*Passer domesticus*	House sparrow		Resident	Nestling
AMR 0086	*Passer domesticus*	House sparrow		Resident	Nestling
AMR 0096	*Passer domesticus*	House sparrow		Resident	Adult
AMR 0110	*Passer domesticus*	House sparrow		Resident	Nestling
AMR 0111	*Passer domesticus*	House sparrow		Resident	Nestling
AMR 0113	*Passer domesticus*	House sparrow		Resident	Nestling
AMR 0116	*Passer domesticus*	House sparrow		Resident	Juvenile
AMR 0121	*Passer domesticus*	House sparrow		Resident	Adult
AMR 0126	*Passer domesticus*	House sparrow		Resident	Juvenile
AMR 0128	*Passer domesticus*	House sparrow		Resident	Juvenile
AMR 0131	*Passer domesticus*	House sparrow		Resident	Nestling
AMR 0134	*Passer domesticus*	House sparrow		Resident	Nestling
AMR 0141	*Passer domesticus*	House sparrow		Resident	Nestling
AMR 0158	*Passer domesticus*	House sparrow		Resident	Adult
AMR 0186	*Passer domesticus*	House sparrow		Resident	Juvenile
AMR 0212	*Passer domesticus*	House sparrow		Resident	Juvenile
AMR 0214	*Passer domesticus*	House sparrow		Resident	Juvenile
AMR 0215	*Passer domesticus*	House sparrow		Resident	Nestling
AMR 0224	*Passer domesticus*	House sparrow		Resident	Juvenile
AMR 0226	*Passer domesticus*	House sparrow		Resident	Nestling
AMR 0229	*Passer domesticus*	House sparrow		Resident	Juvenile
AMR 0236	*Passer domesticus*	House sparrow		Resident	Juvenile
AMR 0246	*Passer domesticus*	House sparrow		Resident	Adult
AMR 0251	*Passer domesticus*	House sparrow		Resident	Juvenile
AMR 0256	*Passer domesticus*	House sparrow		Resident	Adult
AMR 0259	*Passer domesticus*	House sparrow		Resident	Adult
AMR 0270	*Passer domesticus*	House sparrow		Resident	Juvenile
AMR 0271	*Passer domesticus*	House sparrow		Resident	Juvenile
AMR 0278	*Passer domesticus*	House sparrow		Resident	Adult
AMR 0293	*Passer domesticus*	House sparrow		Resident	Juvenile
AMR 0037	*Asio otus*	Long‐eared owl	3	Resident to short distance	Adult
AMR 0132	*Asio otus*	Long‐eared owl		Resident to short distance	Juvenile
AMR 0136	*Asio otus*	Long‐eared owl		Resident to short distance	Nestling
AMR 0032	*Anas platyrhynchos*	Mallard	21	Resident to short distance	Pullus
AMR 0034	*Anas platyrhynchos*	Mallard		Resident to short distance	Pullus
AMR 0035	*Anas platyrhynchos*	Mallard		Resident to short distance	Pullus
AMR 0036	*Anas platyrhynchos*	Mallard		Resident to short distance	Pullus
AMR 0038	*Anas platyrhynchos*	Mallard		Resident to short distance	Pullus
AMR 0039	*Anas platyrhynchos*	Mallard		Resident to short distance	Pullus
AMR 0040	*Anas platyrhynchos*	Mallard		Resident to short distance	Pullus
AMR 0043	*Anas platyrhynchos*	Mallard		Resident to short distance	Pullus
AMR 0044	*Anas platyrhynchos*	Mallard		Resident to short distance	Pullus
AMR 0045	*Anas platyrhynchos*	Mallard		Resident to short distance	Pullus
AMR 0046	*Anas platyrhynchos*	Mallard		Resident to short distance	Pullus
AMR 0047	*Anas platyrhynchos*	Mallard		Resident to short distance	Pullus
AMR 0048	*Anas platyrhynchos*	Mallard		Resident to short distance	Pullus
AMR 0049	*Anas platyrhynchos*	Mallard		Resident to short distance	Pullus
AMR 0050	*Anas platyrhynchos*	Mallard		Resident to short distance	Pullus
AMR 0051	*Anas platyrhynchos*	Mallard		Resident to short distance	Pullus
AMR 0052	*Anas platyrhynchos*	Mallard		Resident to short distance	Pullus
AMR 0078	*Anas platyrhynchos*	Mallard		Resident to short distance	Juvenile
AMR 0155	*Anas platyrhynchos*	Mallard		Resident to short distance	Adult
AMR 0181	*Anas platyrhynchos*	Mallard		Resident to short distance	Pullus
AMR 0193	*Anas platyrhynchos*	Mallard		Resident to short distance	Juvenile
AMR 0001	*Poecile palustris*	Marsh tit	1	Resident	Juvenile
AMR 0234	*Turdus viscivorus*	Mistle thrush	1	Short distance	Juvenile
AMR 0070	*Cygnus olor*	Mute swan	1	Resident	Nestling
AMR 0200	*Milvus milvus*	Red kite	2	Resident to short distance	Juvenile
AMR 0203	*Milvus milvus*	Red kite		Resident to short distance	Juvenile
AMR 0104	*Netta rufina*	Red‐crested pochard	1	Resident to short distance	Juvenile
AMR 0233	*Turdus philomelos*	Song thrush	3	Short distance	Adult
AMR 0267	*Turdus philomelos*	Song thrush		Short distance	Juvenile
AMR 0295	*Turdus philomelos*	Song thrush		Short distance	Juvenile
AMR 0027	*Muscicapa striata*	Spotted flycatcher	2	Long distance	Adult
AMR 0183	*Muscicapa striata*	Spotted flycatcher		Long distance	Nestling
AMR 0249	*Strix aluco*	Tawny owl	1	Resident	Nestling
AMR 0148	*Ciconia ciconia*	White stork	2	Short‐ to long distance	Juvenile
AMR 0184	*Ciconia ciconia*	White stork		Short‐ to long distance	Nestling
AMR 0058	*Motacilla alba*	White wagtail	3	Short distance	Juvenile
AMR 0177	*Motacilla alba*	White wagtail		Short distance	Juvenile
AMR 0216	*Motacilla alba*	White wagtail		Short distance	Nestling
AMR 0292	*Emberiza citrinella*	Yellowhammer	1	Resident to short distance	Adult
AMR 0164	*Larus michahellis*	Yellow‐legged gull	4	Resident to short distance	Nestling
AMR 0188	*Larus michahellis*	Yellow‐legged gull		Resident to short distance	Juvenile
AMR 0202	*Larus michahellis*	Yellow‐legged gull		Resident to short distance	Juvenile
AMR 0280	*Larus michahellis*	Yellow‐legged gull		Resident to short distance	Adult

Note

AMR: antimicrobial resistant.

## References

[mbo3845-bib-0001] Agnew, A. , Wang, J. , Fanning, S. , Bearhop, S. , & McMahon, B. J. (2015). Insights into antimicrobial resistance among long distance migratory east Canadian high arctic light‐bellied brent geese (*Branta bernicla hrota)* . Irish Veterinary Journal, 69(1), 13 10.1186/s13620-016-0072-7 27651892 PMC5024491

[mbo3845-bib-0002] Arnold, K. E. , Williams, N. J. , & Bennett, M. (2016). “Disperse abroad in the land”: The role of wildlife in the dissemination of antimicrobial resistance. Biology Letters, 12, 20160137 10.1098/rsbl.2016.0137 27531155 PMC5014016

[mbo3845-bib-0003] Atterby, C. , Börjesson, S. , Ny, S. , Järhult, J. D. , Byfors, S. , & Bonnedahl, J. (2017). ESBL‐producing *Escherichia coli* in Swedish gulls‐a case of environmental pollution from humans. PLoS ONE, 12, e0190380 10.1371/journal.pone.0190380 29284053 PMC5746268

[mbo3845-bib-0004] Bai, L. , Zhao, J. , Gan, X. , Wang, J. , Zhang, X. , Cui, S. , … Xu, J. (2016). Emergence and diversity of *Salmonella enterica* serovar Indiana isolates with concurrent resistance to ciprofloxacin and cefotaxime from patients and food‐producing animals in China. Antimicrobial Agents and Chemotherapy, 60, 3365–3371. 10.1128/AAC.02849-15 27001808 PMC4879380

[mbo3845-bib-0005] Blane, B. , Brodrick, H. J. , Gouliouris, T. , Ambridge, K. E. , Kidney, A. D. , Ludden, C. M. , … Peacock, S. J. (2016). Comparison of 2 chromogenic media for the detection of extended‐spectrum β‐lactamase producing Enterobacteriaceae stool carriage in nursing home residents. Diagnostic Microbiology and Infectious Disease, 84, 181–183. 10.1016/j.diagmicrobio.2015.11.008 26712266 PMC4769092

[mbo3845-bib-0006] Bonnedahl, J. , Drobni, P. , Johansson, A. , Hernandez, J. , Melhus, A. , Stedt, J. , … Drobni, M. (2010). Characterization, and comparison, of human clinical and black‐headed gull (*Larus ridibundus*) extended‐spectrum beta‐lactamase‐producing bacterial isolates from Kalmar, on the southeast coast of Sweden. Journal of Antimicrobial Chemotherapy, 65, 1939–1944. 10.1093/jac/dkq222 20615928

[mbo3845-bib-0007] Cantón, R. , González‐Alba, J. M. , & Galán, J. C. (2012). CTX‐M enzymes: Origin and diffusion. Frontiers in Microbiology, 3, 110 10.3389/fmicb.2012.00110 22485109 PMC3316993

[mbo3845-bib-0008] Clermont, O. , Christenson, J. K. , Denamur, E. , & Gordon, D. M. (2013). The Clermont *Escherichia coli* phylotyping method revisited: Improvement of specificity and detection of new phylogroups. Environmental Microbiology Reports, 5, 58–65. 10.1111/1758-2229.12019 23757131

[mbo3845-bib-0009] Clinical and Laboratory Standards Institute . (2017). Performance standards for antimicrobial susceptibility testing (27th ed.). CLSI supplement M100S. Wayne, PA: Clinical and Laboratory Standards Institute.

[mbo3845-bib-0010] Federal Office of Public Health and Federal Food Safety and Veterinary Office . (2018). Swiss antibiotic resistance report 2018. Usage of antibiotics and occurrence of antibiotic resistance in bacteria from humans and animals in Switzerland. FOPH publication number: 2018‐OEG‐87, Berne, Switzerland Retrieved from http://www.anresis.ch/index.php/anresisch-data-de.html

[mbo3845-bib-0011] Guenther, S. , Aschenbrenner, K. , Stamm, I. , Bethe, A. , Semmler, T. , Stubbe, A. , … Wieler, L. H. (2012). Comparable high rates of extended‐spectrum‐beta‐lactamase‐producing *Escherichia coli* in birds of prey from Germany and Mongolia. PLoS ONE, 7, e53039 10.1371/journal.pone.0053039 23300857 PMC3534101

[mbo3845-bib-0012] Guenther, S. , Ewers, C. , & Wieler, L. H. (2011). Extended‐spectrum beta‐lactamases producing *E. coli* in wildlife, yet another form of environmental pollution? Frontiers in Microbiology, 2, 246 10.3389/fmicb.2011.00246 22203818 PMC3244693

[mbo3845-bib-0013] Guenther, S. , Grobbel, M. , Lübke‐Becker, A. , Goedecke, A. , Friedrich, N. D. , Wieler, L. H. , & Ewers, C. (2010). Antimicrobial resistance profiles of *Escherichia coli* from common European wild bird species. Veterinary Microbiology, 144, 219–225. 10.1016/j.vetmic.2009.12.016 20074875

[mbo3845-bib-0014] Hernandez, J. , Johansson, A. , Stedt, J. , Bengtsson, S. , Porczak, A. , Granholm, S. , … Bonnedal, J. (2013). Characterization and comparison of extended‐spectrum β‐lactamase (ESBL) resistance genotypes and population structure of *Escherichia coli* isolated from Franklin's gulls (*Leucophaeus pipixcan*) and humans in Chile. PLoS ONE, 8, e76150 10.1371/journal.pone.0076150 24098774 PMC3786981

[mbo3845-bib-0015] Jazmati, N. , Hein, R. , & Hamprecht, A. (2016). Use of an enrichment broth improves detection of extended‐spectrum‐beta‐lactamase‐producing Enterobacteriaceae in clinical stool samples. Journal of Clinical Microbiology, 54, 467–470. 10.1128/JCM.02926-15 26607984 PMC4733169

[mbo3845-bib-0016] Kluytmans‐Van Den Bergh, M. F. Q. , Verhulst, C. , Willemsen, L. E. , Verkade, E. , Bonten, M. J. M. , & Kluytmans, J. A. J. W. (2015). Rectal carriage of extended‐spectrum‐beta‐lactamase‐producing Enterobacteriaceae in hospitalized patients: Selective preenrichment increases yield of screening. Journal of Clinical Microbiology, 53, 2709–2712. 10.1128/JCM.01251-15 25994164 PMC4508429

[mbo3845-bib-0017] Mohsin, M. , Raza, S. , Schaufler, K. , Roschanski, N. , Sarwar, F. , Semmler, T. , … Guenther, S. (2017). High prevalence of CTX‐M‐15‐Type ESBL‐producing *E. coli f*rom migratory avian species in Pakistan. Frontiers in Microbiology, 8, 2476 10.3389/fmicb.2017.02476 29312186 PMC5733096

[mbo3845-bib-0018] Müller, A. , Stephan, R. , & Nüesch‐Inderbinen, M. (2016). Distribution of virulence factors in ESBL‐producing *Escherichia coli* isolated from the environment, livestock, food and humans. Science of the Total Environment, 541, 667–672. 10.1016/j.scitotenv.2015.09.13 26437344

[mbo3845-bib-0019] Oteo, J. , Mencía, A. , Bautista, V. , Pastor, N. , Lara, N. , González‐González, F. , … Campos, J. (2018). Colonization with Enterobacteriaceae‐producing ESBLs, AmpCs, and OXA‐48 in wild avian species, Spain 2015–2016. Microbial Drug Resistance, 24, 932–938. 10.1089/mdr.2018.0004 29782210

[mbo3845-bib-0020] Rebelo, A. R. , Bortolaia, V. , Kjeldgaard, J. S. , Pedersen, S. K. , Leekitcharoenphon, P. , Hansen, I. M. , … Hendriksen, R. S. (2018). Multiplex PCR for detection of plasmid‐mediated colistin resistance determinants. Eurosurveillance, 23, 6 10.2807/1560-7917.ES.2018.23.6.17-00672 PMC5824125 29439754

[mbo3845-bib-0021] Tchesnokova, V. L. , Rechkina, E. , Larson, L. , Ferrier, K. , Weaver, J. L. , Schroeder, D. W. , … Fang, F. C. (2019). Rapid and extensive expansion in the United States of a new multidrug‐resistant *Escherichia coli* clonal group, sequence type 1193. Clinical Infectious Diseases, 68, 334–337. 10.1093/cid/ciy525 29961843 PMC6321845

[mbo3845-bib-0022] Valenza, G. , Werner, M. , Eisenberger, D. , Nickel, S. , Lehner‐Reindl, V. , Höller, C. , & Bogdan, C. (2019). First report of the new emerging global clone ST1193 among clinical isolates of extended‐spectrum β‐lactamase (ESBL)‐producing *Escherichia coli* from Germany. Journal of Global Antimicrobial Resistance. 10.1016/j.jgar.2019.01.014 30682563

[mbo3845-bib-0023] Vangchhia, B. , Abraham, S. , Bell, J. M. , Collignon, P. , Gibson, J. S. , Ingram, P. R. , … Gordon, D. M. (2016). Phylogenetic diversity, antimicrobial susceptibility, and virulence characteristics of phylogroup F *Escherichia coli* in Australia. Microbiology, 162, 1904–1912. 10.1099/mic.0.000367 27666313

[mbo3845-bib-0024] Wirth, T. , Falush, D. , Lan, R. , Colles, F. , Mensa, P. , Wieler, L. H. , … Achtman, M. (2006). Sex and virulence in *Escherichia coli*: An evolutionary perspective. Molecular Microbiology, 60, 1136–1151. 10.1111/j.1365-2958.2006.05172.x 16689791 PMC1557465

[mbo3845-bib-0025] Woodford, N. , Fagan, E. J. , & Ellington, M. J. (2006). Multiplex PCR for rapid detection of genes encoding CTX‐M extended‐spectrum β‐lactamases. Journal of Antimicrobial Chemotherapy, 57, 154–155. 10.1093/jac/dki412 16284100

[mbo3845-bib-0026] World Health Organization . (2014). Global report on surveillance. Geneva, Switzerland: WHO Press Retrieved from https://apps.who.int/iris/bitstream/handle/10665/112642/9789241564748_eng.pdf;jsessionxml:id=E748F6CB85FB55891F79FA5AAB5E7296?sequence=1

[mbo3845-bib-0027] Xia, L. , Liu, Y. , Xia, S. , Kudinha, T. , Xiao, S.‐N. , Zhong, N.‐S. , … Zhuo, C. (2017). Prevalence of ST1193 clone and IncI1/ST16 plasmid in *E. coli* isolates carrying *bla* _CTX‐M‐55_ gene from urinary tract infections patients in *China* . Scientific Reports, 2017, 44866 10.1038/srep44866 PMC5364490 28338012

[mbo3845-bib-0028] Zhang, J. , Zheng, B. , Zhao, L. , Wei, Z. , Ji, J. , Li, L. , & Xiao, Y. (2014). Nationwide high prevalence of CTX‐M and an increase of CTX‐M‐55 in *Escherichia coli* isolated from patients with community‐onset infections in Chinese county hospitals. BMC Infectious Diseases, 14, 659 10.1186/s12879-014-0659-0 25466590 PMC4265337

[mbo3845-bib-0029] Zurfluh, K. , Hächler, H. , Nüesch‐Inderbinen, M. , & Stephan, R. (2013). Characteristics of extended‐spectrum β‐lactamase‐ and carbapenemase‐producing Enterobacteriaceae isolates from rivers and lakes in Switzerland. Applied and Environment Microbiology, 79, 3021–3026. 10.1128/AEM.00054-13 PMC3623138 23455339

[mbo3845-bib-0030] Zurfluh, K. , Nüesch‐Inderbinen, M. , Stephan, R. , & Hächler, H. (2013). Higher‐generation cephalosporin‐resistant *Escherichia coli* in feral birds in Switzerland. International Journal of Antimicrobial Agents, 41, 296–297. 10.1016/j.ijantimicag.2012.11.005 23305658

[mbo3845-bib-0031] Zurfluh, K. , Wang, J. , Klumpp, J. , Nüesch‐Inderbinen, M. , Fanning, S. , & Stephan, R. (2014). Vertical transmission of highly similar *bla* _CTX‐M‐1_‐harboring IncI1 plasmids in *Escherichia coli* with different MLST types in the poultry production pyramid. Frontiers in Microbiology, 5, 519 10.3389/fmicb.2014.00519 25324838 PMC4179741

